# Assessments of arterial and venous phase radiodensity does not improve carotid near-occlusion diagnostics

**DOI:** 10.1038/s41598-024-68732-w

**Published:** 2024-08-10

**Authors:** Karolina Brunnander, Alexander Henze, Allan J. Fox, Elias Johansson

**Affiliations:** 1 Clinical Science, Umeå University, Umeå, Sweden; 2 Radiation Sciences, Umeå University, Umeå, Sweden; 3 Sunnybrook Health Science Centre, University of Toronto, Toronto, ON Canada; 4 Wallenberg Center for Molecular Medicine, Umeå University, Umeå, Sweden; 5 Neuroscience and Physiology, Gothenburg University, Blå Stråket 7, 413 45 Gothenburg, Sweden

**Keywords:** Carotid stenosis, Carotid near-occlusion, Computed tomography angiography, Biphasic, Neurology, Stroke

## Abstract

The hypothesis of this study was that evaluation of radiodensity assessment beyond a carotid stenosis in arterial and/or venous phase can be used to separate near-occlusion and conventional ≥ 50% stenosis. We prospectively included participants with ≥ 50% carotid stenosis with inclusion preference for cases with extracranial internal carotid artery (ICA) asymmetry. All participants were examined with a research biphasic computed tomography angiography (CTA) protocol (arterial and venous phase). Reference diagnosis was set by interpretation on CTA and radiodensity difference between ipsilateral and contralateral ICA (c-corrected) or vertebral (v-corrected) was compared. We included 93 participants, 62 with near-occlusion and 31 with conventional ≥ 50% stenosis. Just beyond the stenosis, median c-corrected radiodensity was − 20 Hounsfield units (HU) among near-occlusions and − 1 HU among conventional ≥ 50% stenoses (*p* < 0.001) in the arterial phase. For the venous phase, these findings were + 17 HU and + 3 HU (*p* = 0.007). Similar group differences were seen for v-correction. No parameter had good diagnostic performance, area under the curve ≤ 0.82. With specificity set at ≥ 95%, detected near-occlusions were foremost those with large side-to-side differences in distal ICA-diameter. Carotid near-occlusions can have reduced radiodensity beyond the stenosis in arterial phases and increased radiodensity in venous phases compared to a reference artery—which was not clearly seen for conventional stenoses. However, these radiodensity findings are best seen in near-occlusion cases that are not diagnostically challenging, while they work poorly as additional diagnostic aids.

## Introduction

Carotid near-occlusion is a variant of severe carotid stenosis with the stenosis associated with a collapse of the internal carotid artery (ICA) beyond the stenosis^1,2^. In contrast, conventional stenoses are not associated with distal collapse. Near-occlusions can be subdivided into those with and without full collapse, depending on the degree of the distal ICA diameter reduction^2^. Recent vascular surgery guidelines recommend routine revascularization for symptomatic conventional ≥ 50% stenoses but revascularization only for symptomatic near-occlusions with repeated symptoms despite best medical therapy and after a multidisciplinary review^3^. However, distinguishing conventional ≥ 50% stenosis and near-occlusion can be difficult^4^. Indeed, recent stroke guidelines has no management recommendation for near-occlusion given diagnostic uncertainties^5^.

Near-occlusion has been diagnosed with a systematic feature interpretation approach in several relevant studies^4^ but seems to not be generally used in routine practice^6^. It may be too difficult for routine practice use^4^. Commonly used ultrasound methods are not accurate as most near-occlusions have high stenosis velocity^7^. Attempts to use CTA diameter measurements have not been accurate when assessed in a blinded manner^4,8^. However, in-our experience, near-occlusions examined in arterial phase occasionally have visibly reduced radiodensity (darker) in the extracranial ICA beyond the stenosis compared to contralateral ICA at same cervical level but increased (brighter) when examined in venous phase. These differences are likely caused by reduced ICA flow that results in a contrast lag: The contrast density will be lower in the arterial phase (not reached yet) distal to a near-occlusion compared to the contralateral distal ICA, but higher in the venous phase (not yet washed out by incoming blood). Such reduced ICA flow is known to be closely associated with a reduced distal ICA diameter^9^. However, no study has assessed whether radiodensity assessments can be diagnostically useful to separate near-occlusion and conventional stenosis, in addition to diameter assessments. Also, a single CTA image set is a “snapshot in time”. It is unclear if an arterial phase, a venous phase or both in combination will be preferable for diagnostic use. Doing both arterial and venous phase with the same contrast bolus (biphasic CTA) is therefore reasonable to study.

The hypothesis of this experiment is that contrast radiodensity of biphasic CTA is diagnostically useful to separate slowed flow carotid near-occlusion and conventional ≥ 50% stenosis.

## Materials and methods

### Participants

This prospective study was conducted between March 2018 and February 2022 at the stroke unit and Department of Radiology at the University Hospital of Northern Sweden. We prospectively included consecutive patients with an initial diagnosis of ≥ 50% carotid stenosis or occlusion who could provide informed consent. As we foremost targeted consecutive inclusion of patients with symptomatic stenosis aimed at revascularization and/or any near-occlusion, patients with conventional asymptomatic stenosis and occlusions were not included. Most participants were assessed for possible symptomatic ≥ 50% carotid stenosis. Patients\with known asymptomatic near-occlusion were included.

This biphasic CTA study is a sub-study with a case–control approach. From the larger cohort of consecutive participants, inclusion criterion for this analysis was ≥ 50% carotid stenosis on at least one cervical side. The exclusion criteria were inability to undergo biphasic CTA (kidney failure and contrast allergy) and unclear diagnosis in subsequent study assessments. In addition, we restricted inclusion to cases with conventional stenosis and symmetric extracranial ICAs to not irradiate many control cases. Consecutive patients with extracranial ICA asymmetry of any cause on an initial assessment were included during the entire study period. In addition to this, we included consecutive patients without ICA asymmetry during a shorter period of the study, aiming at 30 controls without near-occlusion (cases with conventional stenosis with either symmetric or asymmetric extracranial ICAs). As this was based on acute clinical assessments with short delay to carotid revascularization, there were significant logistical challenges to get a true consecutive sample.

All participants in this study underwent a research biphasic CTA scan, described as part of the study informed consent. The additional radiation dose was clearly described as part of the consent. The study was approved by the regional ethics board in Umeå, Sweden, in accordance with the declaration of Helsinki.

## Reference diagnosis

The first CTA was used for the reference diagnosis. However, for participants where the diagnosis had changed by the time of the biphasic CTA (such as progression to occlusion), the biphasic CTA was used. All CTA exams used for reference test assessment were assessed by 3 observers (All by EJ, all until mid-2020 by AJF and all after mid-2020 by AH), blinded to each other and biphasic assessment. Cases with disagreement were resolved by consensus discussion. Near-occlusions were diagnosed if the distal ICA was small, and a severe proximal atherosclerotic stenosis was the most appropriate cause. This was determined by systematic feature interpretation, as described elsewhere^6^. In short, a diagnosis was made using a combination of stenosis severity, distal ICA diameter (in mm), ICA ratio (ICA-to-ICA), ECA ratio (ICA-ECA) and assessment of Circle of Willis. Radiodensity of the arteries was not a specific factor for these assessments, although in cases with severe distal collapse, radiodensity differences between arteries were often obvious. Care was taken to not call near-occlusion for stenoses with small distal ICA due to anatomical variance (associated with asymmetric Circle of Willis) coinciding with the side of the stenosis; these were considered conventional stenosis^10^. A 2-sided conservative approach was used, only analyzing cases where either near-occlusion or conventional stenosis were confident. Hence, we excluded participants with uncertain diagnoses: if the distal ICA was small, and uncertain if a stenosis was the most reasonable cause of a small distal ICA. Conventional stenoses were graded by comparing smallest stenosis lumen with distal ICA well beyond the stenosis (North American Symptomatic Carotid Endarterectomy Trial, NASCET, grading)^2^. Occlusion was diagnosed when no contrast was visible distal to the stenosis in both arterial and venous phases.

### Biphase CTA protocol

A single Siemens CT scanner was used for all exams. We used a biphasic CTA protocol as an arterial phase and a delayed phase that approximated a venous phase (and is referred to as the venous phase). The venous phase timing did not attempt to capture peak venous radiodensity but by the time interval from peak radiodensity in arterial phase to when only 50% of the radiodensity increase from baseline remains. This was 6–8 s delay in a pre-study assessment of bolus tracking in the basilar artery of 15 clinical cases with that information readily available. For the study exams, the arterial phase was captured from aorta to vertex (caudo-cranial direction) and triggered by main contrast bolus arrival in the aorta. The venous phase was then done from below the orbits to C6 vertebral level (cranio-caudal direction). The venous phase scanned a limited field used to catch extracranial ICA, but not deliberately irradiate orbits and thyroid excessively. On the particular machine used, the time to stop table movement after arterial phase, move to the new start position and start the venous phase matched the target of trial runs (6–8 s).

### Radiodensity assessments of biphasic CTAs

**Figure 1:**
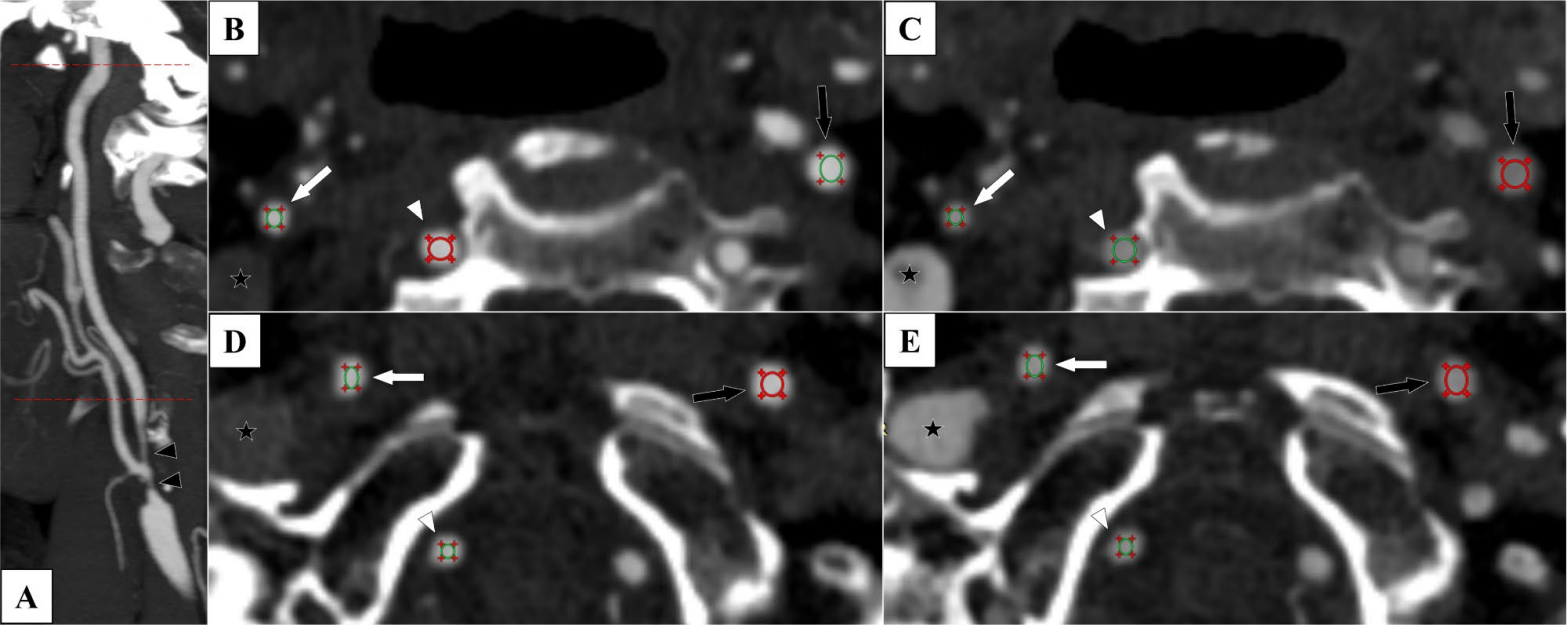
The 12 measurement points in biphasic CTA exams. Case with right-sided near-occlusion without full collapse. 7 s between arterial and venous phase. ROIs placed to avoid the fuzzy edge around the artery. (**A**) Sagittal view with cervical levels (proximal and distal) marked with red dotted lines. Note that the proximal measurement still refers to the artery beyond (distal to) the stenosis (black arrowheads). (**B**) Arterial phase, proximal level. 488, 542 and 535 HU in right ICA (white arrow), left ICA (black arrow) and vertebral (white arrowhead). (**C**) Venous phase, proximal level. 351, 274 and 332 HU. Note the filling of the right jugular vein (black star with white rim). (**D**) Distal level, arterial phase. 486, 521 and 477 HU. Note the non-intuitive change in vertebral HU between proximal and distal phase that shifts the direction of v-correction from − 47 to + 9 HU whereas c-correction is more intuitive (− 54 and − 38 HU). (**E**) Distal level, venous phase. 379, 343 and 386 HU.

There were 12 measurement points for each case in a 3 × 2 × 2 × 2 approach: 3 arteries, 2 sides, 2 levels and 2 phases (Fig. 1). The three arteries were the two ICAs and the visibly largest of the two vertebral arteries. The two levels were: just beyond the stenosis (proximal) and immediately below the entry of the ICA into the skull base (distal). Hence, both proximal and distal level refers to the extracranial ICA beyond the stenosis. The two phases were considered as the arterial and venous phase. A circular region of interest (ROI) was placed and mean radiodensity within the ROI was assessed. The ROI was made as large as possible, but placed and sized so that the area within the ROI was homogenous in color, excluding the fuzzy edge on digital images around the artery lumen. Here, standard deviation (SD) of the Hounsfield units (HUI) within the ROI was measured for quality control, with high SD values signaling non-homogenous sample, prompting an adjusted ROI placement. All radiodensity measurements were done by KB, blinded to clinical information and the reference diagnosis. 18 randomly selected cases were also assessed by EJ to assess inter-rater reliability of radiodensity measurements (each with 12 measurement points, 216 assessments). EJ was also blinded to clinical information and reference diagnosis when performing these assessments.

### Analyses

The ICA with the most severe stenosis was considered the index side regardless of symptomatic side, but occlusions were not preferred. If the stenoses were equally severe on both sides, the index side was assigned randomly. The participants were then divided into near-occlusions or conventional stenoses based on the reference diagnosis assessment on arterial phase CTA. We analyzed absolute values of radiodensity in the 12 measurement points. However, given expected scan-to-scan differences, emphasis was on a corrected ICA value, calculated as index ICA value minus a control measurement point. The control was either the contralateral ICA (c-corrected) or the vertebral (v-corrected) at the same level and same contrast phase. We used subtraction (not division) as absolute difference from 0 HU is irrelevant but would greatly influence a division approach. Also, in order to create a single measurement that includes both arterial and venous phase, a differential value was calculated: The corrected ICA value was expected to be negative in the arterial phase and positive in the venous phase for near-occlusions, and near-zero for conventional stenoses in both phases. Therefore, the differential value was calculated as the corrected ICA in venous phase minus the corrected ICA in the arterial phase. This differential was assessed in a 2 × 2 approach: for both cervical levels and for both c-corrected and v-corrected values.

The approach for the analysis was to first assess group level differences (mean or median) of radiodensity between near-occlusions and conventional stenoses and then assess diagnostic accuracy by receiver-operator curves with thresholds. Thresholds were set for ≥ 95% specificity as high specificity is clinically most useful: Better if a false negative near-occlusion undergo CEA with at most slight net harm than if conventional stenosis is false positive and hence is not subject to much-needed surgery.

It is possible that radiodensity measurements are only diagnostically useful when the blood flow is quite severely reduced, i.e. diagnostically accurate only in near-occlusions with obvious distal collapse, which are not difficult to separate from conventional ≥ 50% stenosis by diameter assessments. Therefore, we assessed how often the high-specificity radiodensity thresholds could correctly classify challenging near-occlusion cases. We did this in two ways: partly by plotting ICA ratio and radiodensity measurements for visual analysis and partly by defining challenging near-occlusion cases as those with ICA ratio ≥ 0.75. This threshold was chosen because the ICA ratio range was 0.75–0.90 among conventional stenoses with ipsilateral smaller distal ICA compared to contralateral side due to anatomical variance.

### Statistics

Where appropriate, we used 95% confidence intervals. Categorical variables were compared with 2-sided χ^2^-test. Continuous variables were considered parametric if skewness and kurtosis was between − 1 and + 1. Parametric variables were presented as mean with SD with 2-sided independent t-test for group comparisons. Non-parametric variables are presented as median with inter-quartile range (IQR) with 2-sided Mann–Whitney for group comparisons. Receiver operator characteristics were calculated with area under the curve (AUC). Inter-rater reliability for CTA assessments was assessed with kappa values for separating conventional ≥ 50% stenosis, near-occlusion and occlusion, assessing both cervical sides. First, for uncertain diagnoses, the cervical sides were counted as conventional ≥ 50% stenosis, then the calculation was repeated with such sides excluded. Inter-rater reliability for radiodensity measurements was assessed with intraclass correlation coefficient, 2-way mixed effects model for absolute agreement. *p* < 0.05 was considered statistically significant and IBM SPSS 28.0 were used for calculations.

## Results

**Figure 2:**
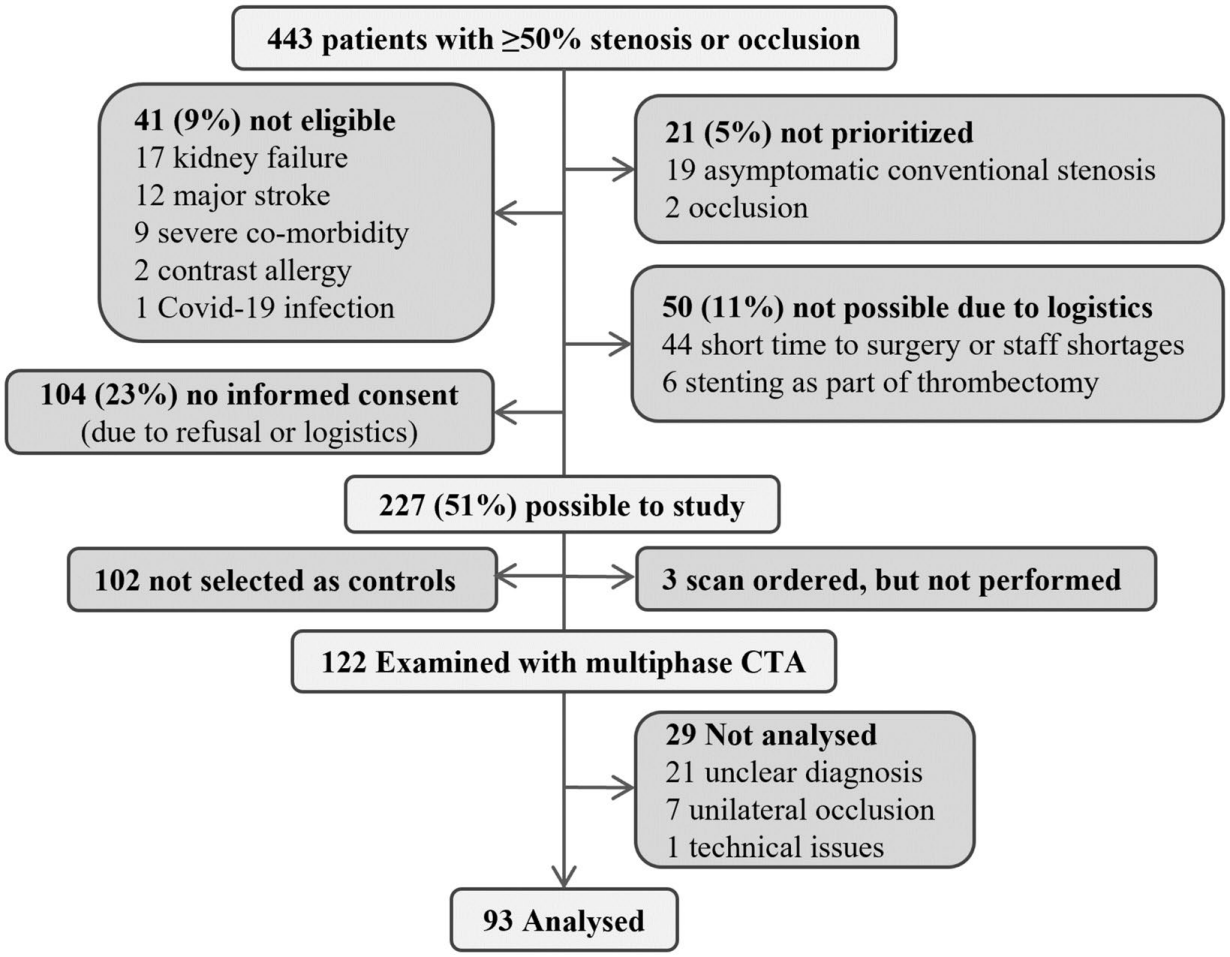
Study flow chart.

Of 444 patients with ≥ 50% stenosis or occlusion, 227 (51%) were possible to study, 122 (28%) were examined with biphasic CTA and 93 (21%) were analyzed, see Fig. 2. Of the 93 participants, 62 had near-occlusions and 31 had conventional stenosis. Among near-occlusions, the mean age was lower and there were more men than among conventional stenoses (Table 1). There were 15 challenging near-occlusion cases, i.e. where ICA ratio overlapped conventional stenoses with anatomical variant. In almost all (85%) of the biphasic exams, the delay between venous and arterial phase was 6 or 7 s, in 10% it was 5 s. After the quality control approach, the radiodensity variation within the ROIs was small: Mean SD in the ROI was between 13 and 18 HU for different measurement points, with > 25 HU in 6% of measurements and no relevant outlier.

**Table 1:** Baseline comparisons.

	Near-occlusion n = 62	Conventional ≥ 50% stenosis n = 31	*p*^a^
Age, mean (SD)	72 (7)	75 (6)	0.019
Women, n (%)	13 (21)	15 (48)	0.009
Symptomatic stenosis, n (%)	52 (84)	28 (90)	0.53
Revascularization, n (%)	45 (73)	26 (84)	0.30
Previous myocardial infarction, n (%)	10 (16)	6 (19)	0.77
Previous revascularization, n (%)	13 (21)	10 (32)	0.31
Current angina, n (%)	5 (8)	2 (6)	1.00
Current smoker, n (%)	11 (18)	4 (13)	0.77
Hypertension, n (%)^b^	54 (87)	27 (87)	1.00
Atrial fibrillation, n (%)	4 (6)	3 (10)	0.68
Heart failure, n (%)	2 (3)	2 (6)	0.60
Diabetes, n (%)	11 (18)	10 (32)	0.19
Ipsilateral stenosis diameter, median (IQR)	0.5 (0.5–0.5)	1.3 (1.1–1.7)	< 0.001
Ipsilateral ICA diameter, (mm)	2.8 (1.8–3.4)	4.2 (3.9–4.7)	< 0.001
Ipsilateral ICA ratio, median (IQR)	0.64 (0.41–0.75)	0.96 (0.87–1.04)	< 0.001
Ipsilateral ECA ratio, median (IQR)	1.0 (0.77–1.2)	1.8 (1.5–2.0)	< 0.001
Ipsilateral conventional ≥ 70% stenosis n (%)	N/A	11 (40)^c^	–
Ipsilateral anatomical variant n (%)^d^	N/A	10 (32)	–
Ipsilateral near-occlusions with full collapse n (%)	14 (23)	N/A	–
Contralateral near-occlusion or occlusion n (%)	5 (8)	5 (16)	0.29

*SD* standard deviation, *IQR* inter-quartile range, *ICA* internal carotid artery, *ECA* external carotid artery, *N/A* not applicable.

^a^2-sided χ^2^-test for categorical, T-test for parametric (presented with mean) and Mann–Whitney for non-parametric (presented with median) parameters.

^b^Blood pressure > 140/90 and/or use of blood pressure lowering medication.

^c^Not assessable in 4 participants due to severe stenosis calcification, although clearly ≥ 50%

^d^ICA asymmetry with smaller ICA on ipsilateral side, caused by Circle of Willis anatomy.

In non-corrected assessments, the mean radiodensity was generally higher for conventional stenoses than near-occlusions in both phases, but this was not always significant in arterial phase (Table 2). This was seen for ipsilateral and contralateral side, indicating the existence of a scan-to-scan effect. Compared to contralateral ICAs, the vertebral arteries generally showed slightly lower HU in arterial phase and higher HU in venous phase.

**Table 2:** Absolute radiodensity measurements in stenosis groups.

Measurement point	Near-occlusion (n = 62)	Conventional ≥ 50% stenosis (n = 31)	*P* (t-test)
Arterial phase			
Proximal index ICA	335 (91)	401 (62)	< 0.001
Distal index ICA	357 (64)	398 (64)	0.06
Proximal contralateral ICA	371 (72)	400 (64)	0.08
Distal contralateral ICA	367 (70)	397 (62)	0.063
Proximal vertebral	364 (63)	385 (53)	0.13
Distal vertebral	345 (60)	355 (64)	0.47
Venous phase			
Proximal index ICA	209 (82)	263 (96)	0.006
Distal index ICA	239 (87)	301 (103)	0.004
Proximal contralateral ICA	190 (74)	249 (88)	0.002
Distal contralateral ICA	219 (82)	287 (97)	0.001
Proximal vertebral	200 (77)	271 (100)	< 0.001
Distal vertebral	239 (81)	312 (107)	< 0.001

*ICA* Internal carotid artery.

All values are mean (SD) for Hounsfield units.

Vertebral: The largest of the two vertebrals was measured.

When correcting for contralateral side (c-correction), near-occlusions had median negative values in the arterial phase and median positive in the venous phase (Table 3); I.e. near-occlusions were darker than the contralateral side for the arterial phase, but brighter in the venous phase. Conventional ≥ 50% stenoses had median generally closer to zero in both phases, i.e., had similar radiodensity on both sides in both phases. There were significant differences between the stenosis groups in all the comparisons except for the proximal ICA venous phase. These differences seemed more pronounced when combining arterial and venous phase measurements (the differential values, Table 3). When correcting for vertebral (v-correction), the group-comparisons were similar as in the c-correction, i.e. near-occlusions had lower HU in arterial phase and higher in venous phase and differential values than conventional stenoses. However, while c-correction resulted in values that followed expectations for above, below or near zero, the values seemed to be more random as to being positive or negative for v-correction (Table 3).

**Table 3:** Group level differences between stenosis groups for radiodensity in index ICA corrected for either contralateral ICA or largest vertebral.

	Near-occlusion (n = 62)	Conventional ≥ 50% stenosis (n = 31)	*P* Mann–Whitney
Contralateral corrected (c-corrected)			
Proximal ICA, arterial phase	− 20 (− 50 to − 4)	− 1 (− 18 to 10)	< 0.001
Distal ICA, arterial phase	− 13 (− 28 to − 1)	− 6 (− 14 to 6)	0.040
Proximal ICA, venous phase	9 (− 4 to 31)	3 (− 10 to 21)	0.22
Distal ICA, venous phase	17 (4–31)	3 (− 11 to 21)	0.007
Differential: Proximal	35 (10–79)	8 (− 14 to 40)	0.002
Differential: Distal	33 (11–55)	4 (− 11 to 37)	< 0.001
Vertebral corrected (v-corrected)			
Proximal ICA, arterial phase	− 7 (− 42 to 7)	16 (4–29)	< 0.001
Distal ICA, arterial phase	17 (− 6 to 29)	36 (22–62)	< 0.001
Proximal ICA, venous phase	5 (− 15 to 23)	− 4 (− 19 to 9)	0.087
Distal ICA, venous phase	− 3 (− 17 to 20)	− 9 (− 35 to 8)	0.17
Differential: Proximal	20 (− 14 to 60)	− 20 (− 47 to 6)	< 0.001
Differential: Distal	− 16 (− 37 to 6)	− 56 (− 71 to − 32)	< 0.001

All values are median (IQR) for Hounsfield units.

Corrections are calculated as index side – control, where the control is either contralateral ICA (c-corrected) or vertebral (v-corrected), both using the same phase and cervical level (proximal or distal) as the index measurement.

Differential: Corrected venous phase – corrected arterial phase. Using the same cervical level.

The highest AUC (0.82) was seen for v-corrected arterial phase, proximal level, but the 95%CI of many AUC assessments overlapped, thus similar (Table 4). The highest sensitivity (51%) at ≥ 95% specificity was seen for ≥ 20 HU v-corrected differential value, proximal level. However, this parameter was positive in 63% (29/46) for the non-challenging near-occluions, but only in 13% (2/15) of the challenging near-occlusions (*p* < 0.001, χ^2^). V-corrected arterial phase, distal level ≤ − 5 HU was seen in 27% (4/15) challenging near-occlusion cases but only in 30% (11/37) of non-challenging near-occlusion. Please refer for to supplemental Fig. 1 for data visualization by scatter plots of all the 12 corrected variables comparisons compared to ICA ratio. The general impression for all parameters were that near-occlusion with radiodensity values clearly different from conventional stenoses had low ICA ratio.

**Table 4:** Diagnostic outcomes of radiodensity measurements to diagnose near-occlusion.

	AUC (95%CI)	Threshold HU^a^	Sensitivity % (n/N)	Specificity % (n/N)	Sensitivity among challenging cases^b^ % (n/N)
Contralateral corrected (c-corrected)					
Proximal ICA, arterial phase	0.72 (0.61–0.84)	≤ − 40	34 (21/62)	97 (30/31)	7 (1/15)
Distal ICA, arterial phase	0.64 (0.52–0.77)	≤ − 31	21 (11/52)	96 (25/26)	7 (1/15)
Proximal ICA, venous phase	0.59 (0.45–0.72)	≥ 46	15 (9/59)	96 (25/26)	0 (0/15)
Distal ICA, venous phase	0.69 (0.55–0.82)	≥ 57	7 (4/54)	96 (25/26)	7 (1/15)
Differential: Proximal	0.71 (0.60–0.82)	≥ 64	32 (19/59)	96 (25/26)	0 (0/15)
Differential: Distal	0.73 (0.60–0.85)	≥ 72	15 (8/52)	96 (25/26)	7 (1/15)
Vertebral corrected (v-corrected)					
Proximal ICA, arterial phase	0.82 (0.73–0.91)	≤ − 15	45 (28/62)	97 (30/31)	7 (1/15)
Distal ICA, arterial phase	0.78 (0.67–0.88)	≤ − 5	28 (15/54)	97 (30/31)	27 (4/15)^c^
Proximal ICA, venous phase	0.61 (0.49–0.73)	≥ 29	18 (11/61)	100 (31/31)^d^	0 (0/15)
Distal ICA, venous phase	0.59 (0.46–0.72)	≥ 32	19 (10/54)	97 (30/31)	13 (2/15)
Differential: Proximal	0.78 (0.68–0.87)	≥ 20	51 (31/61)	97 (30/31)	13 (2/15)
Differential: Distal	0.79 (0.69–0.89)	≥ 8	23 (12/52)	97 (30/31)	13 (2/15)

*AUC* Area Under the Curve, *HU* Hounsfield units, *ICA* Internal carotid artery.

Variable details are as in Table 3.

^a^Set for ≥ 95% specificity.

^b^Challenging case was defined as near-occlusion with ICA ratio ≥ 0.75. This ICA ratio threshold was chosen as conventional stenoses with anatomical variations all had ICA ratio ≥ 0.75.

^c^Sensitivity 30% (11/37) among non-challenging near-occlusions.

^d^If threshold was set at ≥ 28, specificity dropped to 94% without improving sensitivity.

### Inter-rater reliability

The CTA observers had 91% agreement with kappa 0.81 (95%CI 0.73–0.89) for all cervical sides. If excluding cervical sides where either observer assessed it as unclear diagnosis, the CTA observers had 95% agreement with kappa 0.90 (95%CI 0.84–0.97). Intraclass coefficient between observers for 216 radiodensity measurements was 0.88 (95%CI 0.83–0.91).

## Discussion

The main findings in this study were that on group level, near-occlusions have darker distal ICA in arterial phase and brighter in venous phase compared to contralateral distal ICA, but this was not seen for conventional stenoses. However, the diagnostic usefulness of radiodensity findings were poor, especially in challenging near-occlusion cases.

Given group level-findings for c-correction, we can confirm that which previously was anecdotal: The distal ICA is generally darker in near-occlusion than the contralateral side in the arterial phase and brighter in the venous phase. This was not clearly present in conventional stenoses. However, there was considerable overlap between the two groups of stenosis, seen in the overlap in IQR (Table 3), the modest AUCs (Table 4), and in data visualization scatter plots (Supplemental Fig. 1) why the diagnostic usefulness was limited. The ≥ 20 HU v-corrected differential, proximal level was reasonably sensitive (51%) when threshold was set for ≥ 95% specificity. However, this was foremost caused by non-challenging near-occlusions being positive in 63% of cases, whereas challenging near-occlusions were only positive in 13% of cases. While v-corrected arterial phase, distal level was positive in 27% of challenging near-occlusions, the robustness of this finding was questionable as it was only positive in 30% of non-challenging near-occlusions. Hence, our results suggest that when radiodensity assessments are specific for near-occlusions, they are not sensitive. Even worse, for the challenging cases that need additional diagnostic markers, radiodensity performed poorly. While further studies might prove us wrong, it does not seem like radiodensity measurements are the solution to the diagnostic issues of accurate and feasible separation of near-occlusion and conventional stenosis in routine practice. This is disappointing as routine addition of a post-contrast CT head (with distal ICAs assessable)^11^ could have been an easy approach to perform the radiodensity measurements. Rather, phase contrast-MRI has been reported as a much more accurate technique to identify near-occlusions (> 95% sensitive and specific)^9,12^. Assessments of distal ICA velocity for carotid ultrasound^13^ should also be further assessed and there are imaging methods not yet assessed for near-occlusion identification^14^.

Absolute radiodensity values are presented for complete reporting rather than as a diagnostic aid. Large variance in absolute numbers is to be expected between scans, especially for different patients from different CTA studies, dependent upon variables such as neck circumference, differences of cardiac physiological factors, and delayed contrast arrival and clearance due to physiological venous variables including near the clavicle.

The c-corrections resulted in intuitively reasonable findings between groups and for positive, negative or near-zero HU. The v-corrections had also reasonable findings between groups but seemingly random values as to if they were positive, negative or near-zero HU. This could be a random phenomenon or possibly caused by general differences in contrast phases between the carotid and vertebral circulation. Regardless, this non-intuitive pattern would benefit from validation before being trusted. The rationale for adding the v-corrections was to enable corrected values in cases with contralateral ICA occlusion, and we did not expect that v-correction would have improved diagnostic outcomes than c-correction. It should be noted that there are fewer cases with missing data in v-correction than c-correction due to contralateral occlusions (Table 4).

We used a novel definition for “challenging near-occlusions” (ICA ratio > 0.75) in order to assess the likely diagnostic addition of radiodensity assessments to diameter measurements. We didn’t intend for this to be a specific group definition but only as a reasonable pedagogical and research approach to complement the scatterplots presented in data supplement. The rationale was reasonable as it defined the overlap between near-occlusions and anatomical variants. The value (> 0.75) was also reasonable as it is smaller than both a threshold for separating near-occlusion and conventional stenosis (≤ 0.87)^15^, a threshold for when distal ICAs are visibly different (≤ 0.88)^10^, and larger than a suggested definition of full collapse (≤ 0.42)^16^.

Near-occlusion with full collapse might imply to some a high risk of early stroke recurrence, but this was not found in all relevant studies^3,16,17^. If symptomatic near-occlusion with full collapse will be shown to need different management than what is currently recommended for symptomatic near-occlusions; the biphasic approach might be useful to separate near-occlusion with full collapse from occlusion, which can sometimes be difficult^6^. Further studies into this topic are warranted.

Strengths of this study were a prospective approach with a prespecified hypothesis based on anecdotal experience. This hypothesis was tested with a purpose-designed novel biphasic protocol to enable assessment of both arterial and venous phases simultaneously, to remove between-scan variations and assess the possibility that both phases combined could be better than each by its own. We also used state-of-the-art reference test assessments with good inter-rater reliability. While we can show good inter-rater reliability for our systematic interpretation approach between collaborating expert observer, this is likely not applicable in routine practice, hence the need for additional diagnostic aids.

Limitations include a rather small sample size of controls, but this was intentional for ethical reasons (reduce radiation). The approach was analogous to a phase II trial: An initial experience where sample was limited for ethical reasons and cases of interest (near-occlusions and conventional stenosis with anatomical variants) were favored for inclusion similar to the placebo arm is often smaller than all the active treatment arms. Additionally, our experiment used two points in time as serial imaging; more serial points might have given different results. Given our negative results, it seems ethically questionable to continue biphasic assessments for this indication. Assessing readily available arterial phase scans would be ethically unproblematic, but the chance of a relevant positive outcome seems limited. Also, we excluded all cases with unclear cause of small distal ICA, similar to some previous studies.

## Conclusion

Overall, near-occlusions can have lower radiodensity in the distal ICA in arterial phases and higher radiodensity in venous phases reflecting delayed flow, compared to contralateral distal ICA, whereas conventional ≥ 50% stenoses usually have symmetric radiodensity in both phases. However, due to the overlap between the groups, radiodensity measurements from this experiment have limited diagnostic value and virtually no additional values when taking an easy metric such as the ICA ratio into account.

### Supplementary Information

Supplementary Figures.

Supplementary Legends.

## Data Availability

The datasets generated during and/or analyzed during the current study are available from the corresponding author on reasonable request.
