# Supplementary figures and images for: Assessments of arterial and venous phase radiodensity does not improve carotid near-occlusion diagnostics
Source: Sci Rep. 2024 Aug 10;14:18616. doi: 10.1038/s41598-024-68732-w (PMC11316748; doi:10.1038/s41598-024-68732-w)

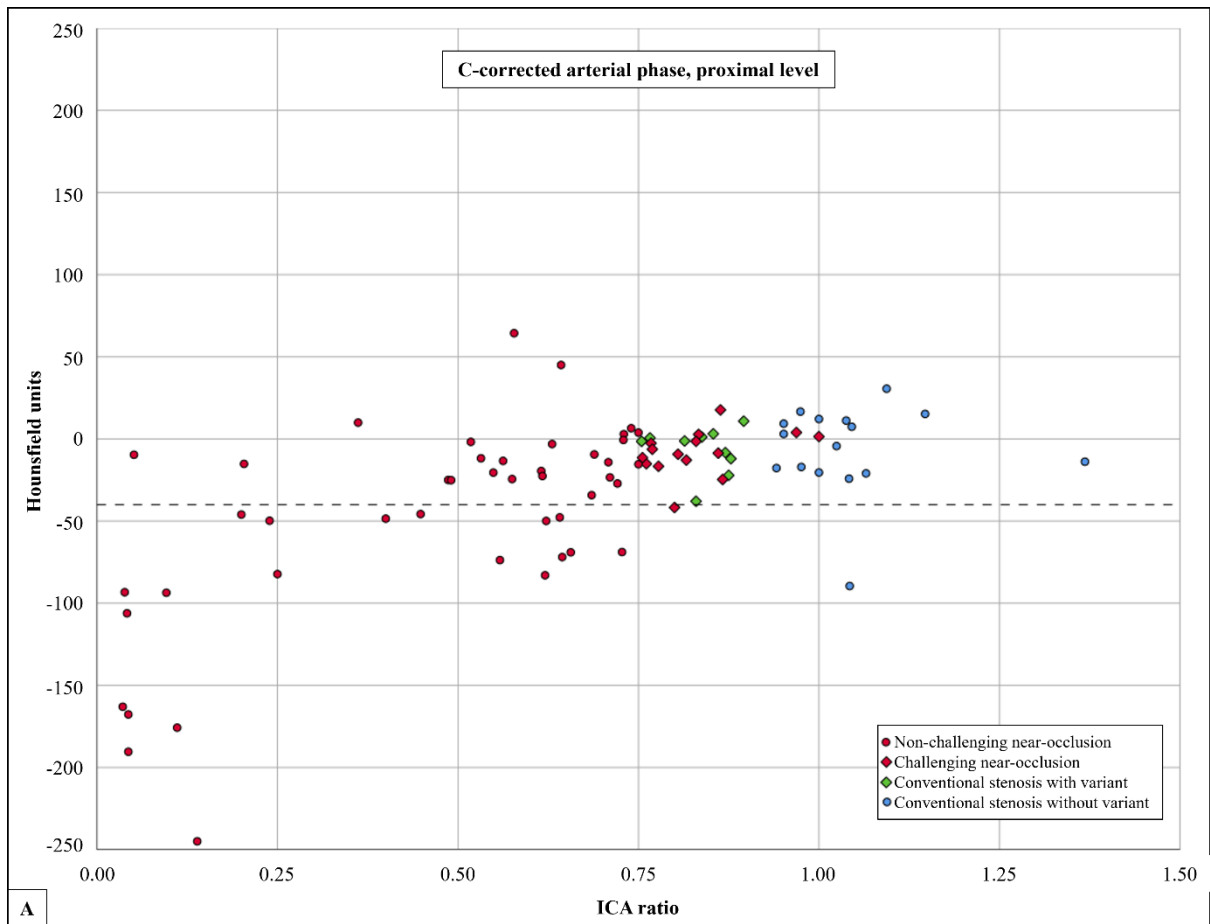

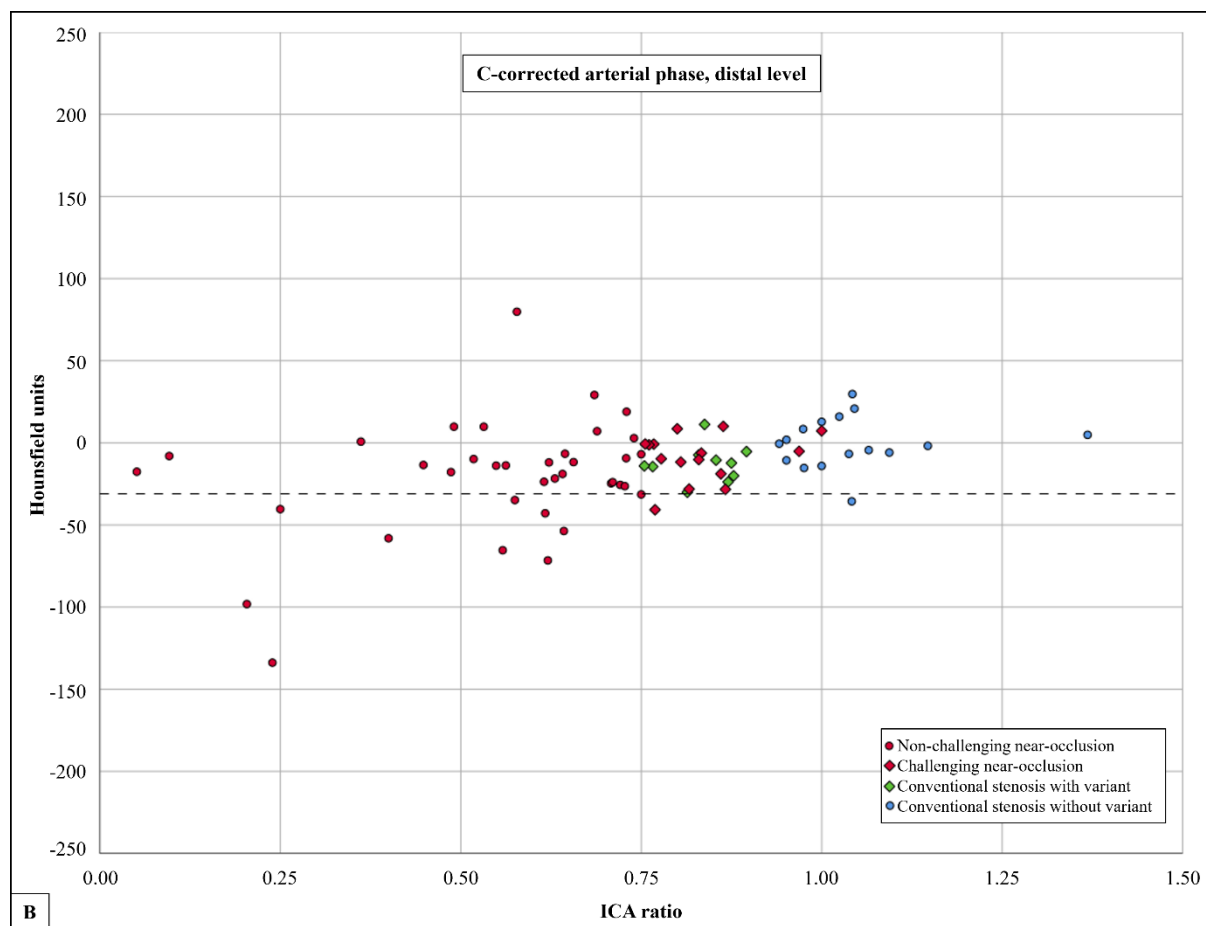

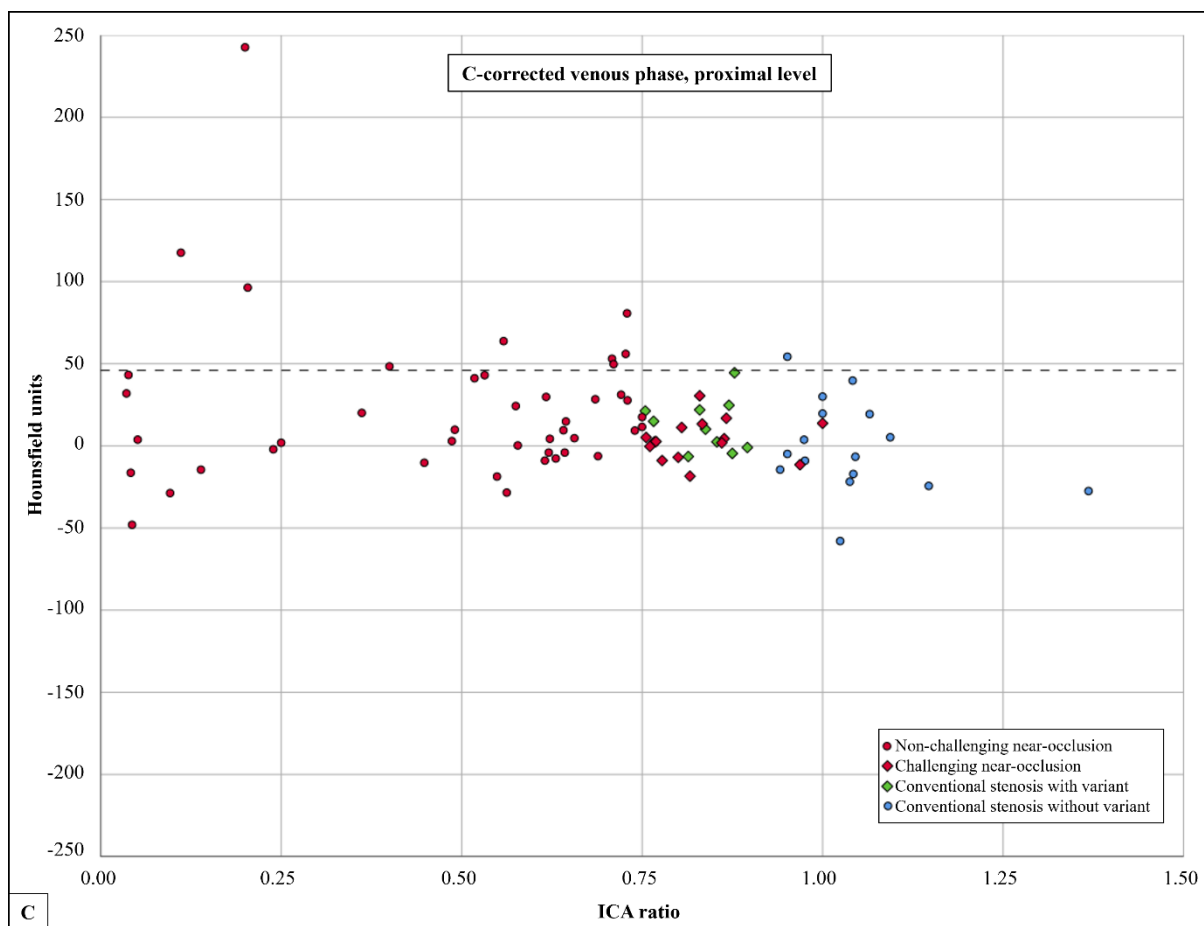

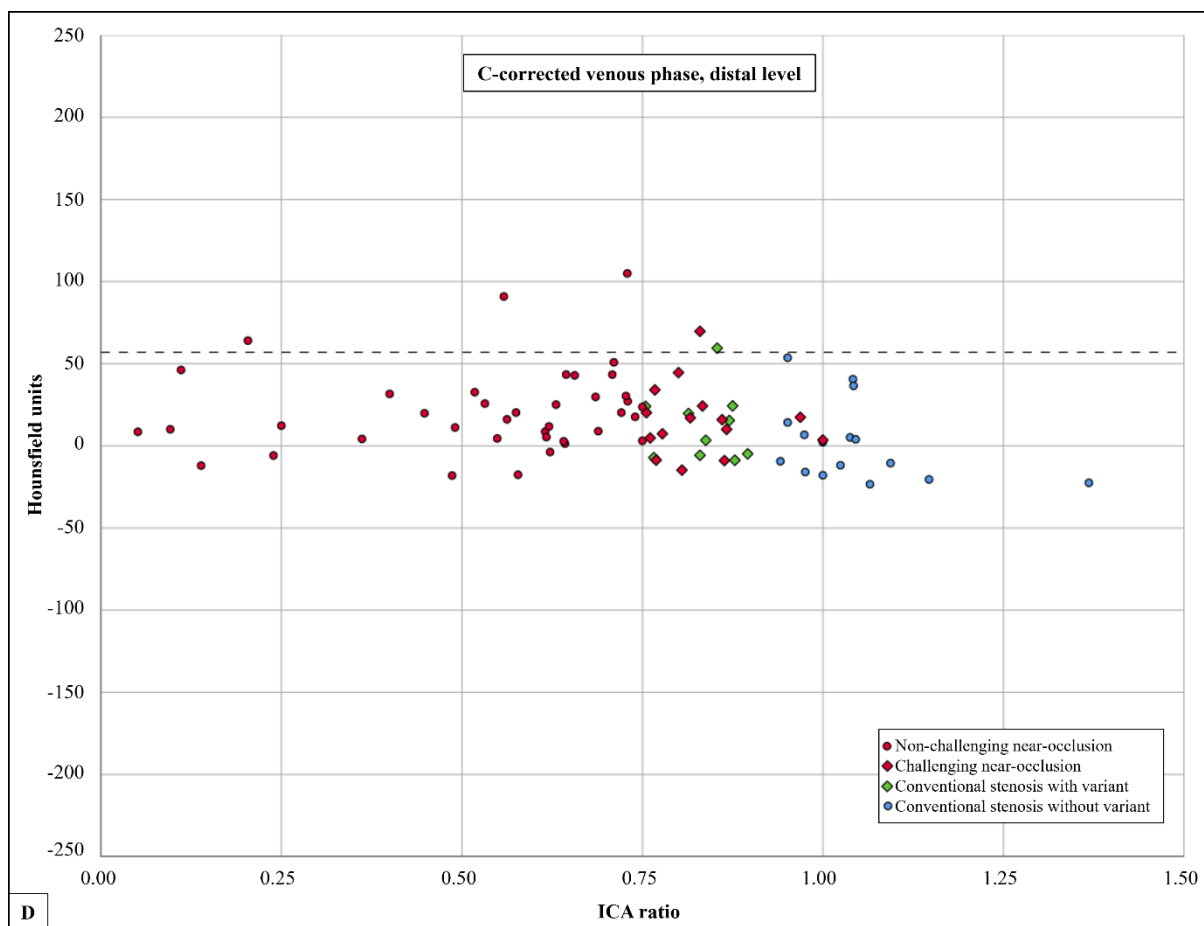

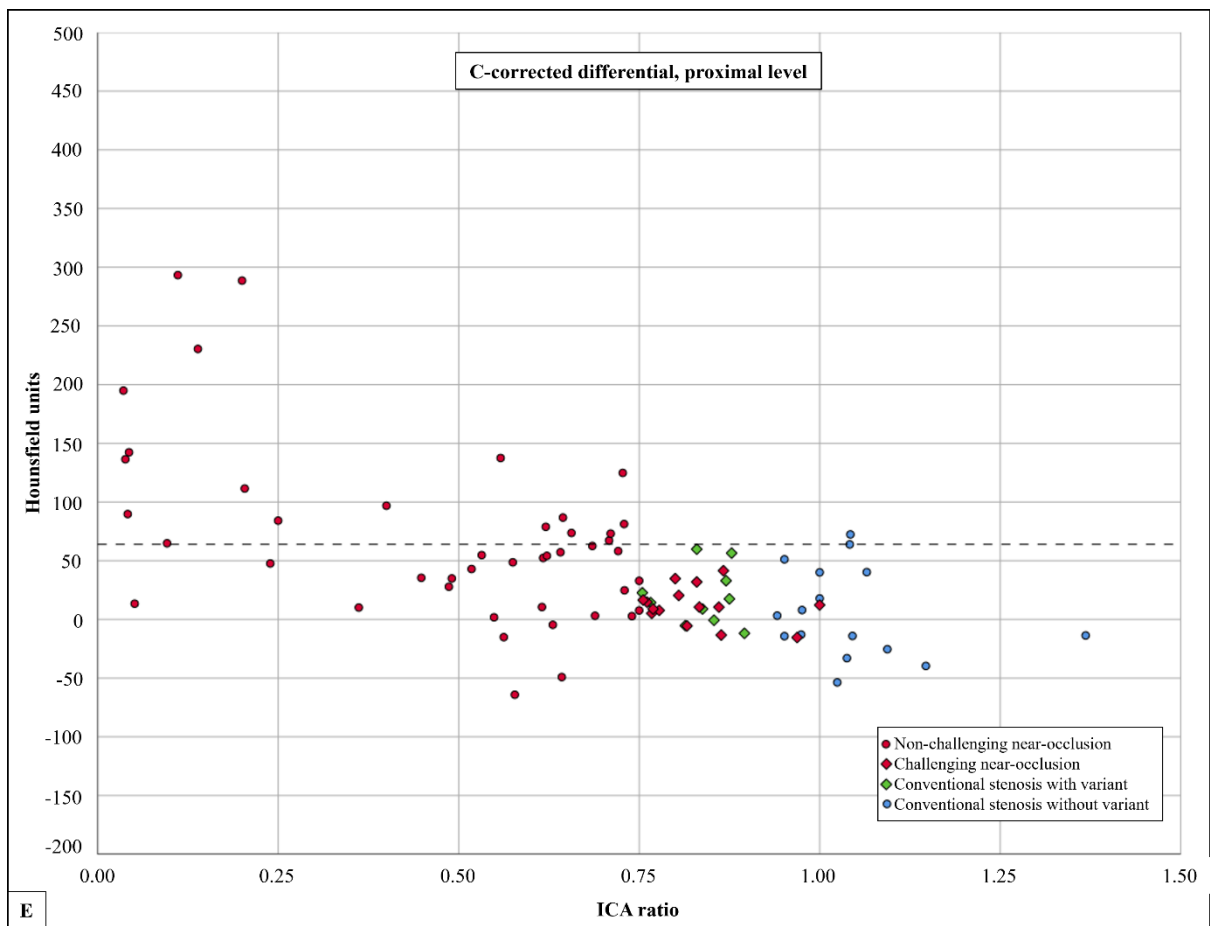

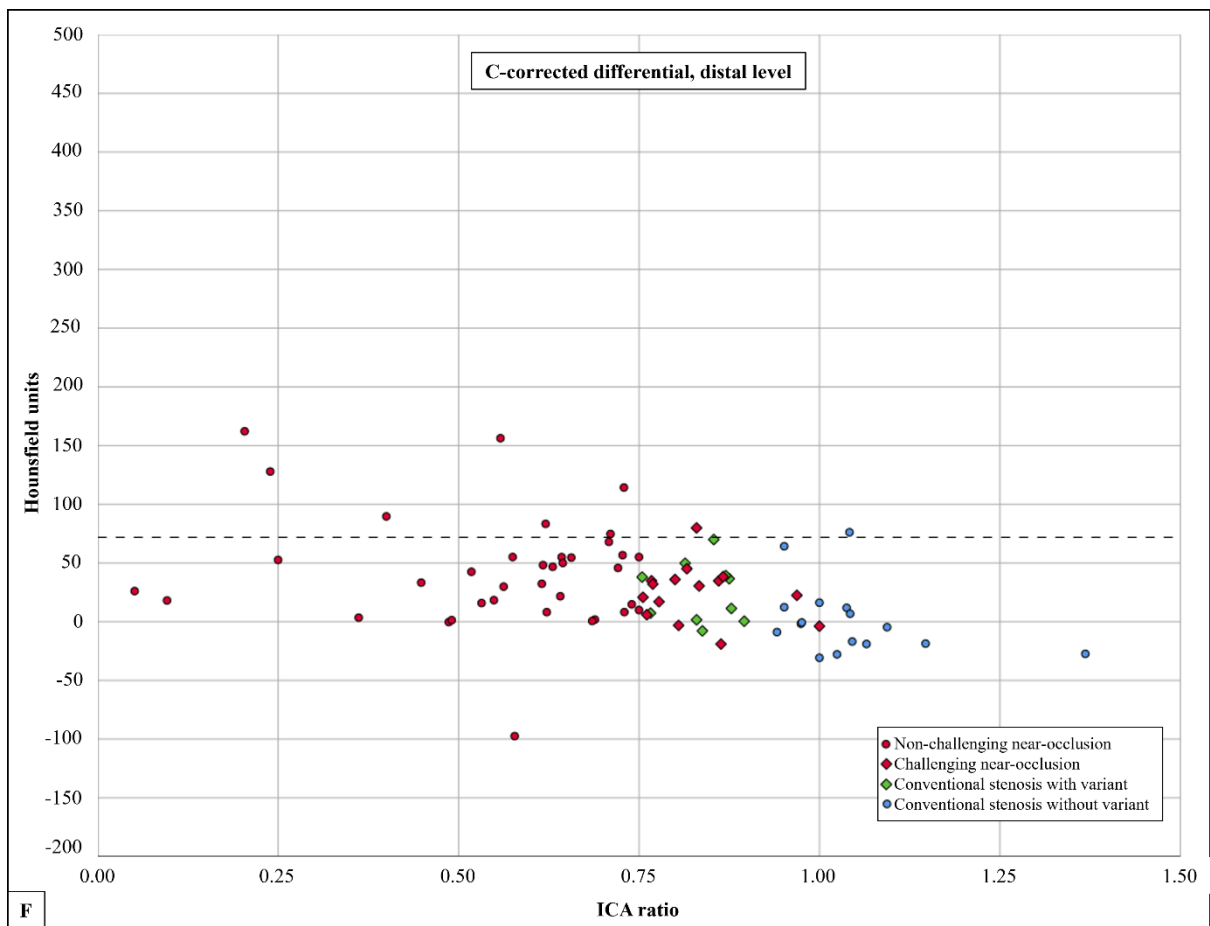

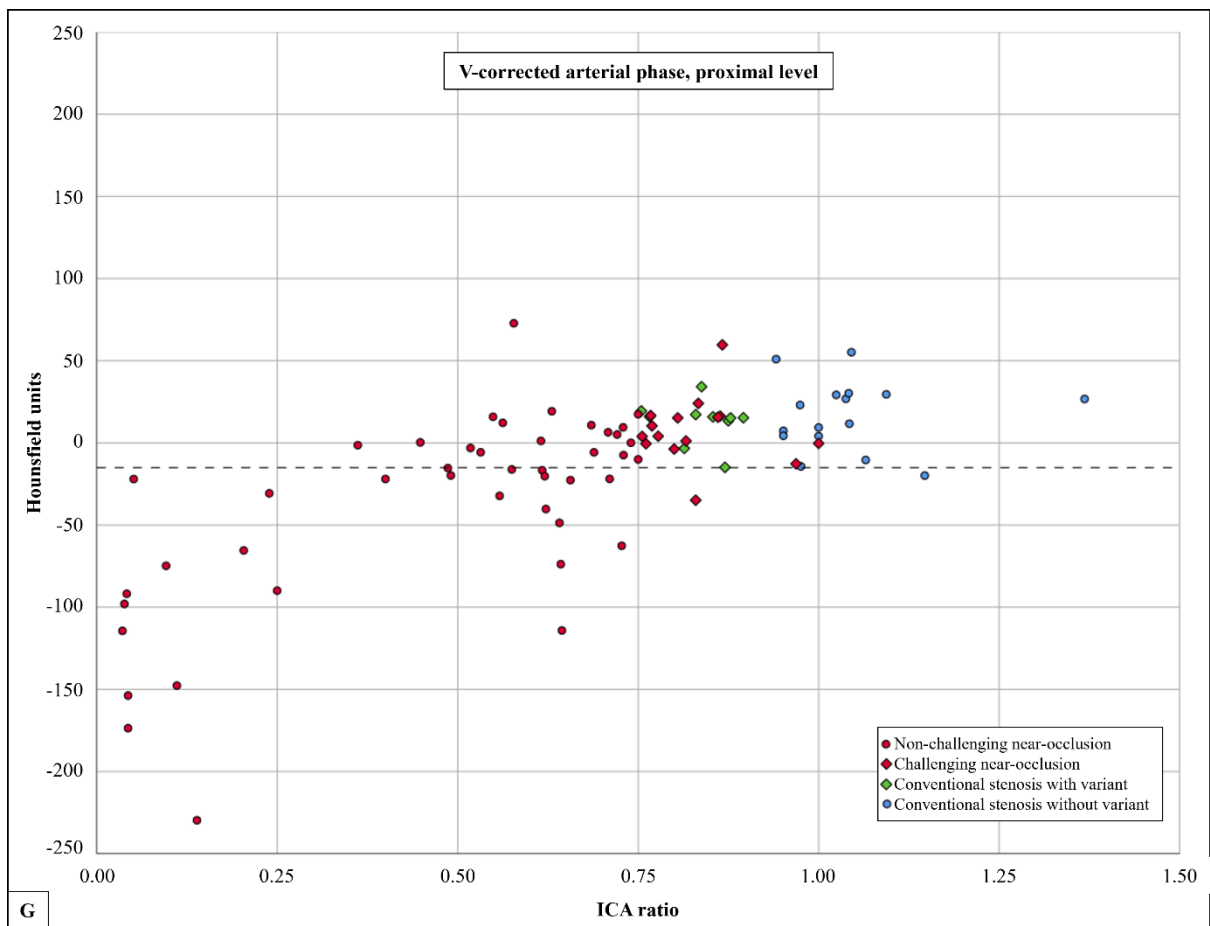

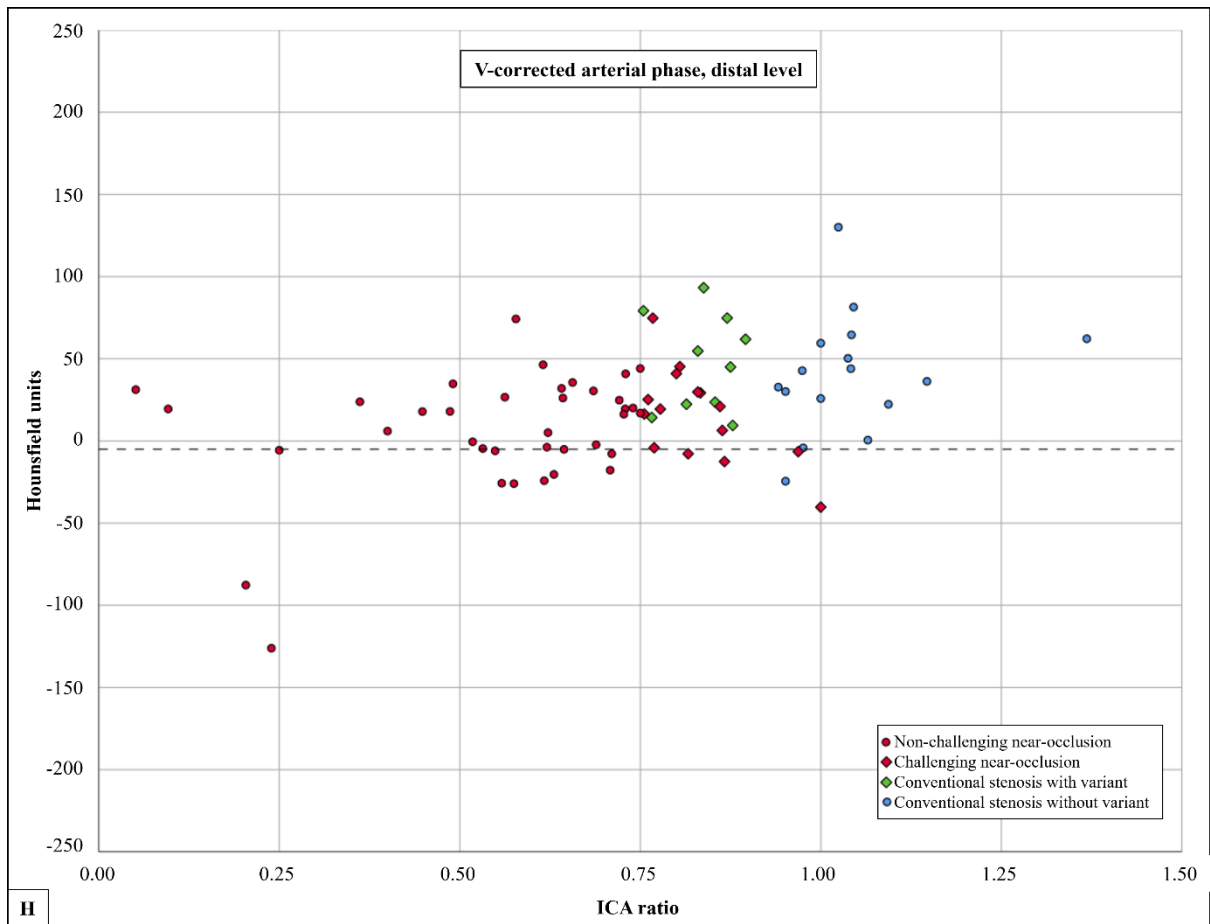

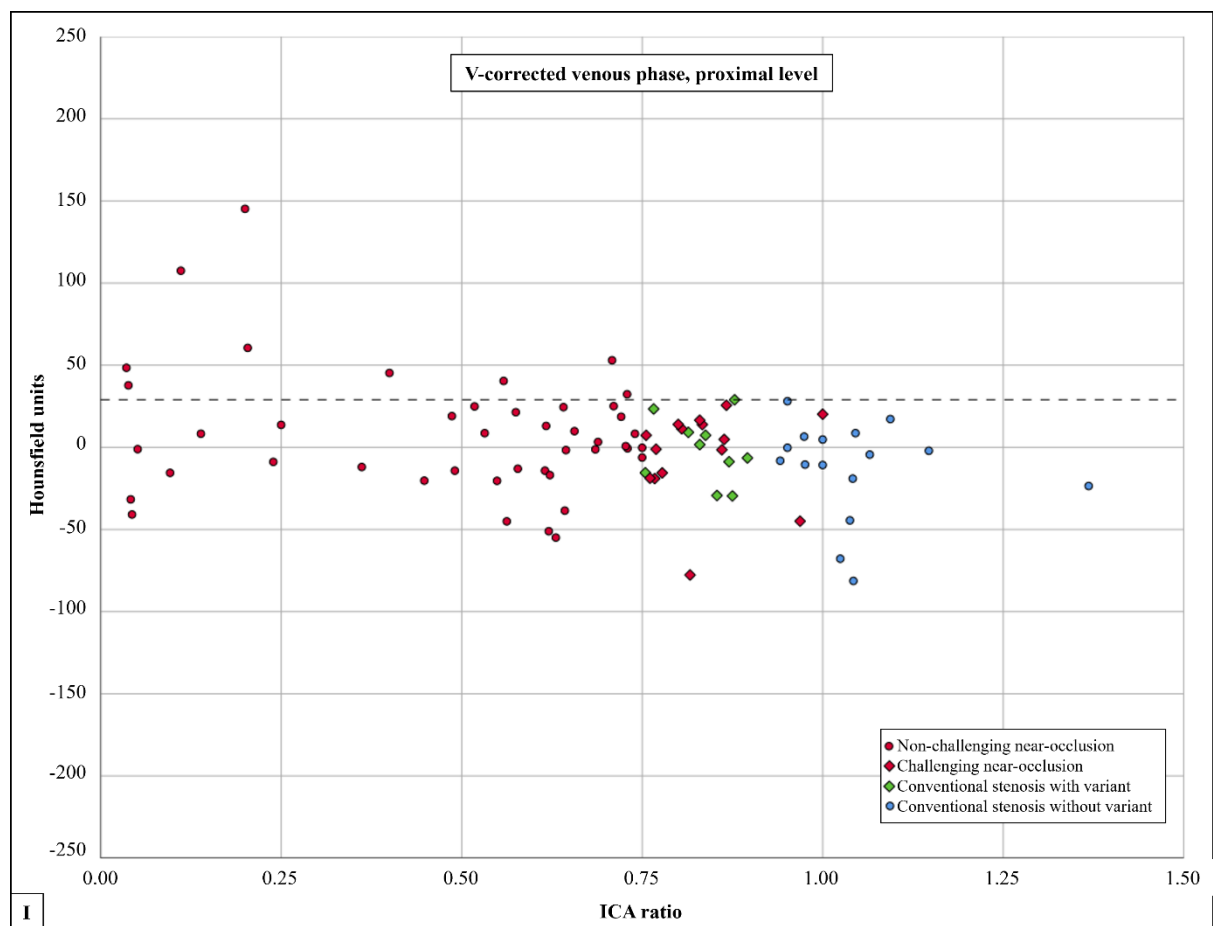

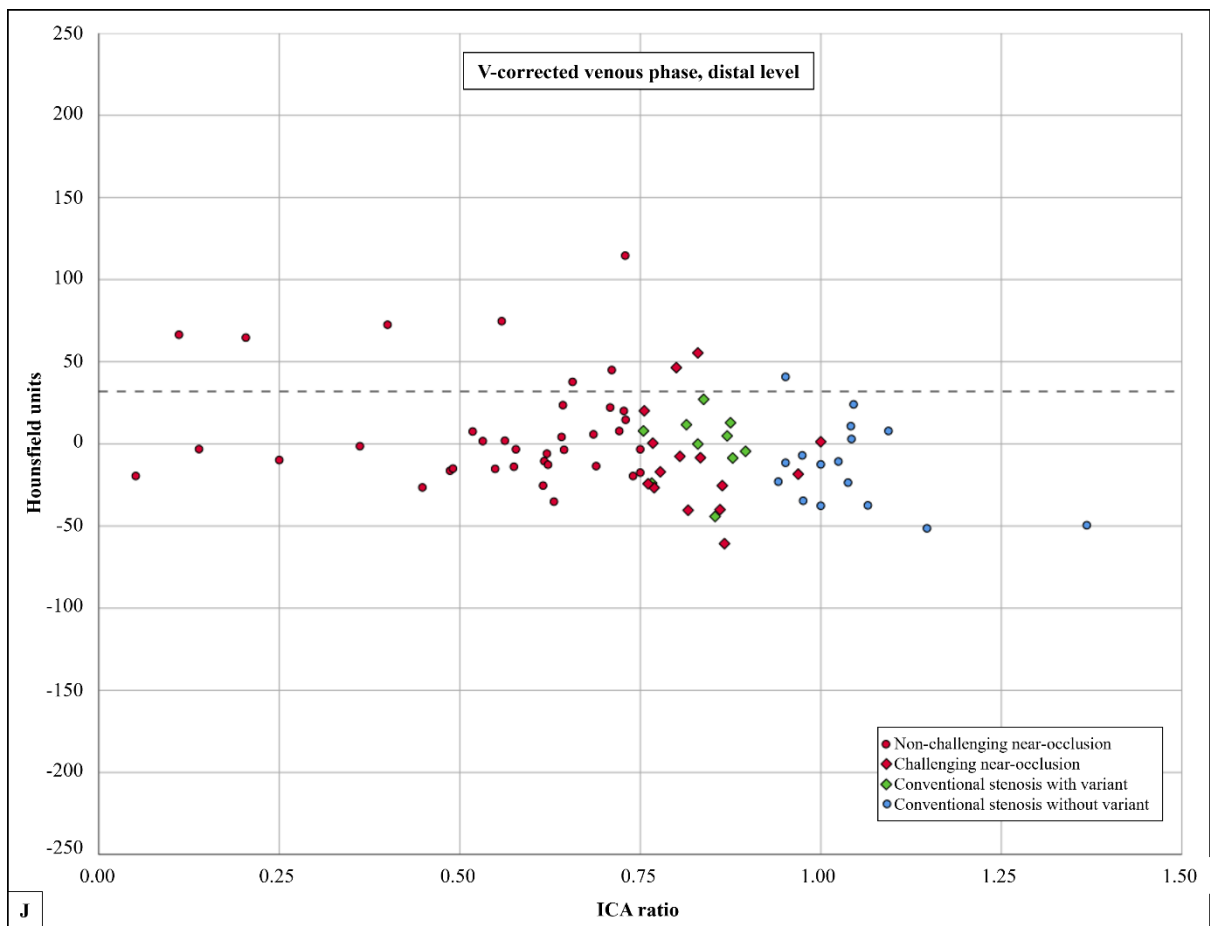

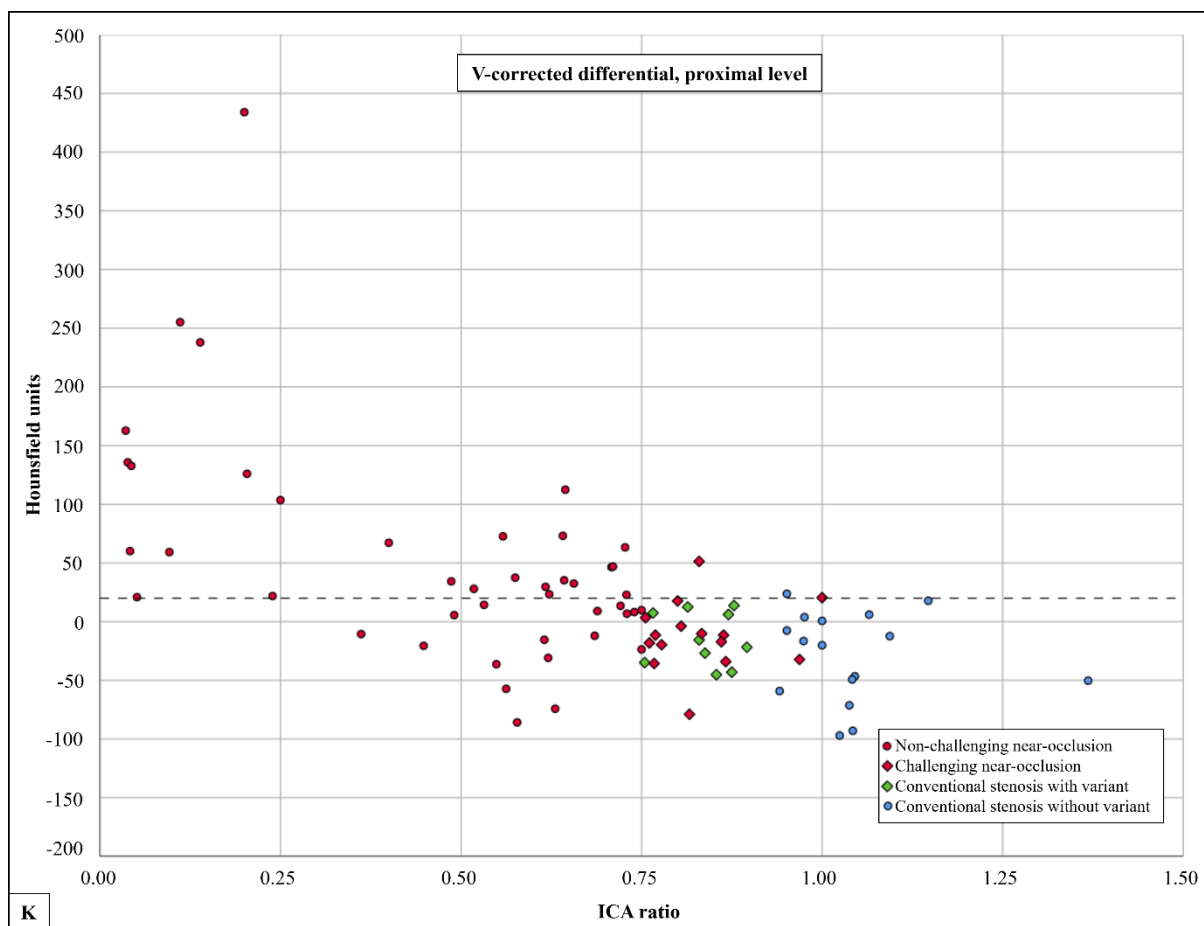

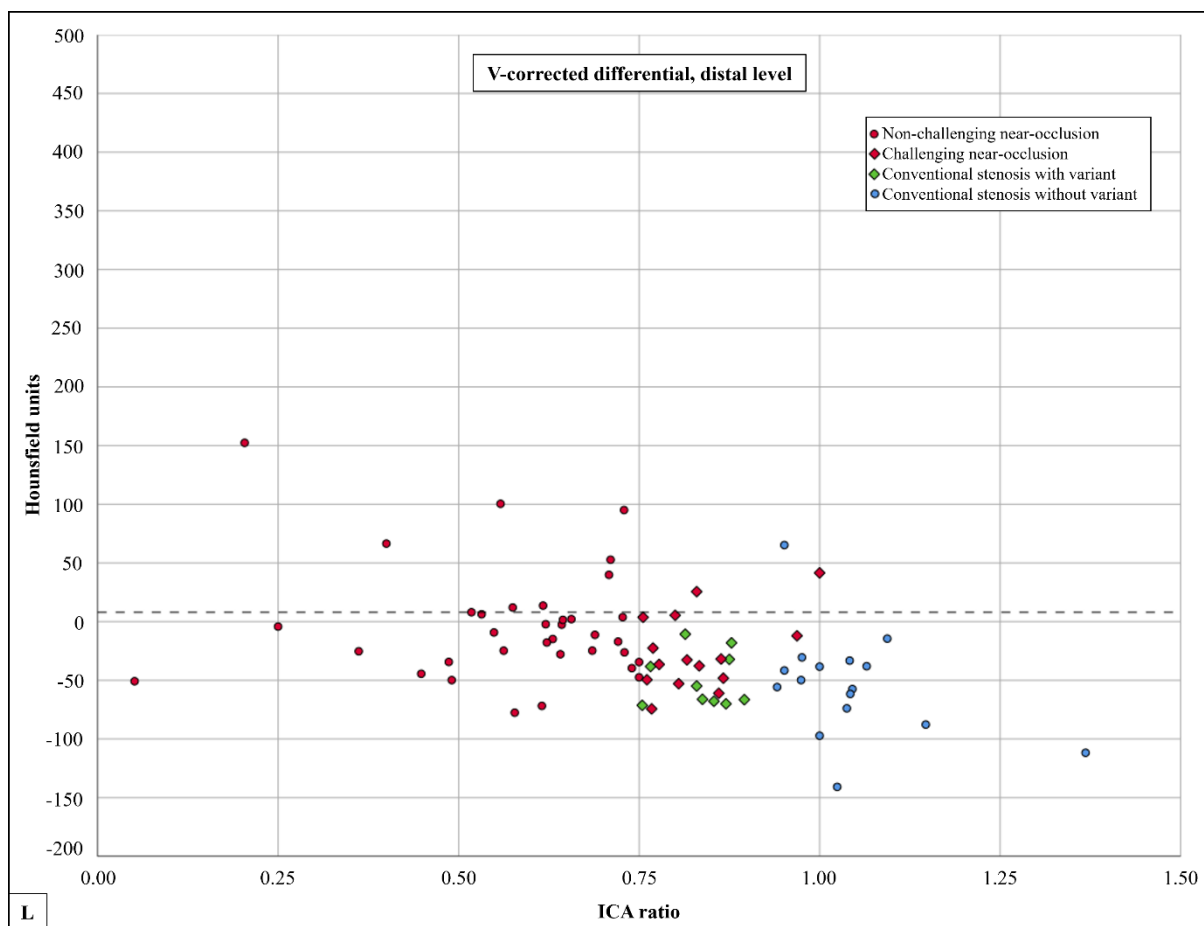

Supplement: Supplementary file 1 — Supplementary Figures. [file 41598_2024_68732_MOESM1_ESM.zip › 41598_2024_68732_MOESM1_ESM.pdf]
