# Supplementary material for: Assessments of arterial and venous phase radiodensity does not improve carotid near-occlusion diagnostics
Source: Sci Rep. 2024 Aug 10;14:18616. doi: 10.1038/s41598-024-68732-w (PMC11316748; doi:10.1038/s41598-024-68732-w)
Supplement: Supplementary file 2 — Supplementary Legends. [file 41598_2024_68732_MOESM2_ESM.pdf]

Assessments of arterial and venous phase radiodensity does not improve carotid near-occlusion diagnostics

Supplemental figure 1

Scatterplots to visualize the outcomes. Dashed line marks the threshold for ≥95% specificity (table 4). A-F) C-corrected. G-L) V-corrected. Note difference in Y-axis scale between differential (E,F,K,L) and the remaining. L) Seeminlgly misplaced specificity-line was caused by a case with conventional stenosis and 7 HU that is not visualized due to contralateral occlusion (has no ICA ratio).
